# Development and validation of HPTLC fingerprints of three species of *Alpinia* with biomarker Galangin

**DOI:** 10.1186/s12906-017-2033-4

**Published:** 2018-01-16

**Authors:** Anuradha S. Upadhye, Anagha Rajopadhye, Lourelle Dias

**Affiliations:** 1 Biodiversity and Palaeobiology, Agharkar Research Institute, G.G. Agarkar Road, Pune, 411004 India; 2 Sinhgad College of Science, S.No. 9/1/5 & 9/2/4, Off West. Bypass Highway, Ambegaon, Pune, Maharashtra 411041 India

**Keywords:** *Alpinia* sp., Adulterants or substitutes, Galangin, Hptlc

## Abstract

**Background:**

*Alpinia galanga* (L.) Willd*.* commonly called as *Rasna*, *Greater galangal* or *Kulinjan* is a medicinally important rhizome used in Indian traditional system of medicine to cure a number of ailments. *A. galanga* is the main source of a galangin -a medicinally important flavanol which has a number of pharmacological properties viz. anti-mutagenic, and anti-inflammatory. Due to the high demand for the rhizome of *A. galanga* traders are now substituting it with rhizomes of *A. calcarata* and *A. officinarum.*

**Methods:**

The present study aims to develop high performance thin layer chromatographic (HPTLC) fingerprinting of *A. galanga* with its adulterants or substitutes and to quantify bioactive galangin present thereof. Methanolic extracts were obtained from rhizomes of the three species of *Alpinia* used for HPTLC analysis using silica gel 60 F254 plates and hexane: ethyl acetate: acetic acid (6.2: 2.8: 1.0 *v*/*v*/*v*); the densitometric analysis was performed at 272 nm.

**Results:**

By comparison of R*f* values and of the spectra of the bands with those of the standard galangin was identified in all three samples. HPTLC quantitative analysis of the methanolic extracts showed the decline trend in the quantity of the galangin in the three species of *Alpinia* as *A. galanga* (7.67 ± 0.36 mg/g) > *A. officinarum* (5.77 ± 0.71 mg/g) > *A. calcarata* (4.31 ± 0.44 mg/g). The HPTLC method was validated using International Conference on Harmonization (ICH) guidelines. The HPTLC method showed good linearity, recovery and high precision of biomarker.

**Conclusions:**

Rapid and reproducible method is useful for routine analysis of galangin and quality control of *Alpinia galangal* along with its adulterants or substitutes.

## Background

Genus *Alpinia* consist of 230 species from family Zingiberaceae [1]. One of these, rhizome of *Alpinia galanga* (L.) Willd*.* is used in Indian traditional system of medicine for rheumatism, fever, bronchial catarrh, stomach pain, stimulant, carminative, tonic, aphrodisiac, aromatic and to decrease the urine output in diabetic patients etc. [2]. It is commonly known as *Greater galangal* in English, *Rasna* in Sanskrit and traded as *Kulinjan* in Indian market [3]. It is native to Eastern Himalaya and distributed in China, Malaya, Indonesia, Thailand, India [4] Philippines and Indonesia [5].

Rhizomes of *A. galanga* are reported for anti-microbial, anti-diabetic [6], anti-inflammatory, anti-cancer [7], anti-flatulence [8], anti-fungal in AIDS patients [9], cytoprotective [10] and anti-allergic activity [11]. The chemical study reported presence of a wide array of bioactive phytoconstituents in the rhizome of *A. galanga* as galangin, α-pinene, β-pinene, limonene, cineol, terpinen-4-ol, α-terpineol, resin containing galangol, kaempferide, methyl cinnamate, camphor, myricene, methyl eugenol, flavones, alpinin, 3-deoxy-4-methoxy [12] and 1′-acetoxychavicol acetate [13]. Of which, it is a foremost source of flavonol galangin having the diverse source of biological and pharmacological properties, such as anti-mutagenic, anti-clastogenic, antioxidative, anti-inflammatory [14], metabolic enzyme modulating, anti-proliferative and anticancer activity [15].

Crude drug samples of *A. galanga* commonly known as ‘*Kulinjan*’ were studied for their biological resource. Owing to its high demand in the Indian market, *A. galanga* is seen to be adulterated/substituted with rhizomes of two other species *Alpinia calcarata* (Haw.) Roscoe and *Alpinia officinarum* Hance under the common trade name [16, 11]. In early research, *A. calcarata* has been used as a synonym for *A. galanga* [17]. However, as per recent nomenclature, both species are considered as separate entities.

HPTLC fingerprinting with bioactive marker confirms authenticity and quality of herbal medicine. A perusal of literature showed that reports on comparative HPTLC fingerprinting of herb *A. galanga* (rhizome) and its adulterants /substituent with reference to marker compound galangin was not available. Hence, the aim of present study was to develop a comparative HPTLC fingerprint of three species of *Alpinia* and quantification of galangin in the methanolic extract thereof using ICH guidelines [18].

## Methods

### Chemicals and plant materials

Standard galangin (CAS 548–83-4; Purity ≥ 98.0%) was purchased from Fluka-Aldrich Chemical, Steinheim, Germany. Silica gel F254 HPTLC plates were purchased from Merck, Darmstadt, Germany. Other analytical grade solvents and regents were obtained from S. D. Fine chemicals, Mumbai, India.

Rhizomes of *A. galanga* and *A. calcarata* were collected from Dapoli and Naoroji Godrej Centre for Plant Research, Maharashtra, India in October 2013, respectively. The rhizomes of *A.officinarum* were procured from local Pune market in the same year. They were authenticated and deposited at Agharkar Herbarium of Maharashtra Association of Cultivation Science (AHMA) repository, Agharkar Research Institute, Pune 411,004; vide voucher specimen number R-199, R-200 and R-201.

### Preparation of sample solutions

Accurately weighted (10 g) powdered samples were extracted exhaustively with methanol (50 mL) using Soxhlet apparatus with an extraction time of 480 min (8 h). The extracts were concentrated under reduced temperature and pressure using rotary evaporator. Respective yields of methanol extract of samples were *A. galanga* (AGM) - 0.990 g, *A. calcarata* (ACM) - 0.867 g and *A. officinarum* (AOM) -0.9501 g.

### Chromatography

HPTLC was performed on aluminium backed HPTLC plates (10 × 10 cm) coated with 0.2 mm layers of silica gel 60 F254 (E. Merck, Germany). Samples were applied on the plate with band width 6 mm employing Linomat IV sample applicator (Camag, Switzerland) fitted with a microlitre syringe. Linear ascending development of the plates to a distance of 80 mm was performed with mobile phase hexane: ethyl acetate: acetic acid (6.2: 2.8: 1.0 *v*/*v*/*v*) in a twin trough glass chamber previously saturated with mobile phase vapour for 10 min at 25 °C. The dried plate was scanned at the wavelength of 272 nm (λmax of galangin) using a Camag TLC scanner 3 with CATS 4 software.

For the calibration curve, a standard stock solution (1 mg/mL) was prepared by dissolving 1 mg accurately weighed galangin in methanol and diluting it to 10 mL in the volumetric flask. Working standard galangin solutions 3, 6, 9, 12 and 15 μg/mL of different concentrations; 50, 100, 150, 200 and 250 ng, respectively were prepared by diluting the stock solution. Each solution (10 μL) was applied on the plate and the plate was developed under predetermined conditions described above. The procedure was repeated thrice to plot a graph of response (peak area) and amount of galangin.

### Quantification of galangin

**Fig. 1:**
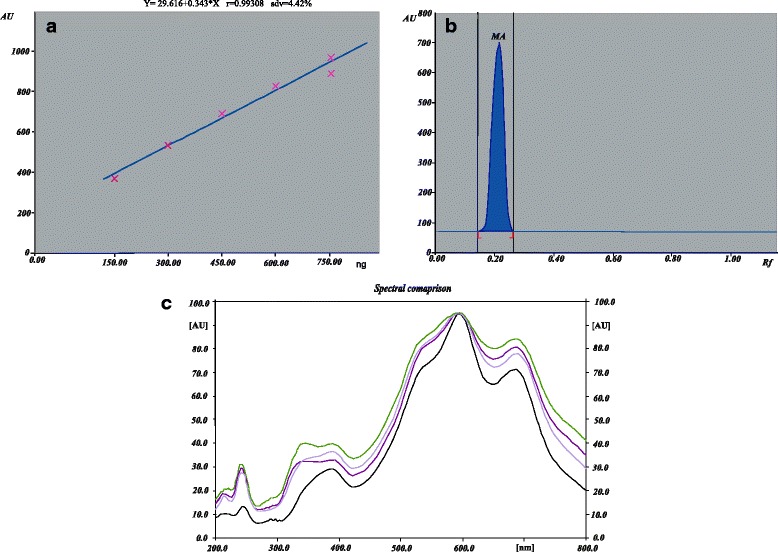
**a** Linear range of calibration plots for galangin; **b** Standard galangin used as a phytomarker; **c** Overlaid spectra of standard galangin and three species of *Alpinia*

Suitably diluted solutions of test samples (10 μL) were applied in triplicate on a HPTLC plate along with standard. The plate was developed under predetermined conditions described above and scanned at 272 nm (λmax of galangin). Peak areas were recorded and galangin content in the samples was calculated using the calibration plot (Fig. 1).

### Validation of the method

The method was validated according to the ICH guidelines [18] by determining peak purity, limit of detection (LOD), limit of quantitation (LOQ), precision and recovery of galangin from the samples. LOD and LOQ were determined by diluting known concentrations of standard stock solution until the average responses were approximately three or ten times the responses of the blank.

Instrument precision was checked by repeated scanning of galangin band (200 ng) six times and was expressed as percent relative standard deviation (% RSD). Precision was studied by analyzing six bands of sample solution per plate on three plates (intra-day precision) and by analyzing six bands of sample solution per plate on three consecutive days (inter-day precision) at three different amount (100, 150 and 200 ng) and calculated % RSD. The accuracy of the method was tested by determination of recovery at three levels. Pre-analyzed samples were spiked with extra galangin (50, 100, and 150%) and the mixtures were reanalyzed. The robustness of the method was studied at three different concentrations, such as 100, 150 and 200 ng per band galangin by introducing small deliberate changes in mobile phase composition hexane: ethyl acetate: acetic acid (6.1: 2.8: 0.9; 6.3: 3: 0.8; 6.0: 3.1: 1.0 *v*/*v*/*v*). The repeatability of the method was assessed by analysis of 120 ng per band of the standard solution of galangin (*n* = 6) and expressed as % RSD of peak areas. Percentage recovery and standard deviation (SD) were calculated for each concentration level. LOD and LOQ were determined by the standard deviation (SD) method from the slope (S) of the calibration plot and the SD of a blank sample (blank methanol was spotted three times), by use of the equations LOD = 3.3 × SD/S and LOQ = 10 × SD/S.

### Chromatographic fingerprinting

Chromatographic fingerprinting of three species of *Alpinia* along with bioactive marker galangin was developed using above chromatographic conditions.

### Statistical analysis

The analysis of each processed extract of three *Alpinia* spp. was carried out in triplicates and results were reported as mean ± SD.

## Results and discussion

Owing to the high automatization, HPTLC technique can be used for the analysis of complex mixtures of natural products. A clear recognition by United States Pharmacopeia [19] and Chinese Pharmacopoeia [20] points out the importance of this technique as the method of choice for handling complex analytical task involving herbal drugs and botanicals.

Herbal drugs are seen to be a mixture of phytoconstituents, in which no single constituent can be attributed for overall efficacy. Thus, it is necessary to establish certain quality control standards for raw materials and herbal products. Quantification of the content of marker present in herbal medicine is proving to be a standard method for evaluating the phytochemical entity of the herb. A flavonol, galangin is a bioactive constituent from *Alpinia* sp. The method will be helpful in determining the amount of galangin in different species of *Alpinia* which will provide significant advantages in terms of greater specificity and rapid analysis.

The mobile phase (hexane: ethyl acetate: acetic acid-6.2: 2.8: 1.0 *v*/*v*/*v*) gave the optimized results with sharp, symmetrical and well-resolved peaks of galangin at R*f* 0.42 from other components of the sample extracts (Fig. 1). A linear relationship was obtained between response (peak area) and amount of galangin in the range of 50–250 ng/band; the correlation coefficient was 0.9919. A biomarker galangin in the methanolic extracts of three species of *Alpinia* was quantitated using the developed HPTLC method. The trend of galangin amount was found to be as *A. galanga* (7.67 ± 0.36 mg/g) > *A. officinarum* (5.77 ± 0.71 mg/g) > *A.calcarata* (4.31 ± 0.44 mg/g).

The method was validated in terms of peak purity, precision, LOD, LOQ and accuracy (Tables 1, 2 and 3) and was specific for analysis of active principle galangin because it resolved at R*f* 0.42 in the presence of other components. Selectivity was shown by applying standard solutions to the plate. The purity of the galangin peak from the sample was confirmed by overlaying UV absorption spectrum of samples with standards at 272 nm. The % RSD of instrument precision for peak area of galangin was found to be 0.72. Inter- and intraday precision were studied by triplicate assay at three different quantities (100, 150, 200 ng per spot). Robustness of the method was indicated by low RSD values calculated of peak areas (Table 2). The accuracy of the method was determined at three levels (50, 100, and 200%) by adding a known amount of standard to the sample extracts. Recovery varied between 93.69 to 97.41%. High recovery indicated that the proposed method was reliable and reproducible (Table 3). LOD and LOQ were 15.15 and 52.63, respectively.

**Table 1:** Validation data of HPTLC method for the estimation of glangin

Intermediate precision (% RSD, *n* = 6)	0.72
Calibration range (ng per spot)	50–250
Regression equation	Y = 0.313 x + 29.319
Correlation coefficient	0.9919
Repeatability of Standards (% RSD, n = 6)	0.91
Repeatability of Samples (% RSD, n = 6)	0.83
Limit of Detection (LOD) (ng per spot)	15.15
Limit of Quantitation (LOQ) (ng per spot)	52.63
Robustness (% RSD, *n* = 3)	0.79

**Table 2:** Study of intra-day and inter-day precision for galangin

Concentration (ng per band)	Intra-day (% *RSD*, *n* = 6)	Inter-day (% *RSD*, *n* = 6)
100	2.89	4.17
150	3.11	4.89
200	1.05	2.27

**Table 3:** Accuracy of the method for galangin

Sample extract	Amount of galangin(ng)^a^			Recovery (%)^a^	Average recovery (%)^a^
	Sample	Added	In mixture		
*A. galanga*	640 ± 20.89	320	948 ± 11.28	98.75	97.41
	640 ± 20.89	640	1260 ± 15.01	96.88	
	640 ± 20.89	960	1855 ± 13.45	96.61	
*A. calcarata*	455 ± 25.66	227.5	623 ± 8.89	91.35	93.69
	455 ± 25.66	455	870 ± 10.03	95.60	
	455 ± 25.66	682	1070 ± 12.21	94.11	
*A. officinarum*	670 ± 15.42	335	941 ± 10.74	96.62	94.37
	670 ± 15.42	670	1263 ± 17.28	94.25	
	670 ± 15.42	1005	1545 ± 23.08	92.24	

^a^Mean ± SD(*n*=3)

A precise and accurate HPTLC technique was developed, which allowed quantitative and qualitative evaluation of galangin in methanolic extracts of three species of *Alpinia*. Mobile phase optimized for HPTLC effectively resolved galangin.

This validity of data along with the HPTLC fingerprints for each individual species may help in correct identification of *Alpinia* spp. of important medicinal utility.

## Conclusions

The rapid and reproducible method developed with HPTLC is useful for routine analysis of galangin and quality control of Alpinia galangal along with its adulterants or substitutes.

## References

[1] Raj G, Pradeep DP, Yusufali C, Dan M, Baby S (2013). Chemical profiles of volatiles in four *Alpinia* species from Kerala, South India. J Essent Oil Res.

[2] Shetty RG, Monisha S (2015). Pharmacology of an endangered medicinal plant *Alpinia galanga* – a review. Res J Pharm Biol Chem Sci.

[3] Anonymous, The Ayurvedic Pharmacopoeia of India. Government of India Ministry of Health and Family Welfare. New Delhi; 2006. 5(1) p.90-92.

[4] Verma RK, Mishra G, Singh P, Jha KK, Khosa RL (2011). Alpinia Galanga – an important medicinal plant: a review. Der Pharmacia Sin.

[5] Yan WU, Ying W, Zhi-Hua LI, Cheng-Fang W, Jian-Yu W, Xiao-Lan LI, Ping-Juan W, Zhao-Feng Z, Shu-Shan DU, Dong-Ye H, Zhi-Wei D (2014). Composition of the essential oil from *Alpinia galanga* rhizomes and its bioactivity on *Lasioderma serricorne*. Bull Insectol.

[6] Jeyachandran R, Mahesh A (2007). Enumeration of antidiabetic herbal flora of Tamil Nadu. Res J Med Plant.

[7] Kale VM, Namdeo AG (2015). HPTLC densitometric evaluation by simultaneous estimation of galangin in *Alpinia galanga* and *Alpinia officinarum*. Der Pharmacia Lett.

[8] Wungsintaweekul J, Sitthithaworn W, Putalun W, Hartwig WP, Brantner A (2010). Antimicrobial, antioxidant activities and chemical composition of selected Thai spices. Songklanakarin J Sci Technol.

[9] Udomkusonsri P, Trongvanichnam K, Klangkaew MLN, Napasorn KKJ (2007). In vitro efficacy of the antifungal activity of some Thai medicinal-plants on the pathogenic fungus, *Saprolegnia parasitica*, from fish. Nat Sci.

[10] Indrayan AK, Agrawal P, Rathi AK, Shatru A, Agrawal NK, Tyagi DK (2009). Nutritive value of some indigenous plant rhizomes resembling ginger. Nat Prod Rad.

[11] Namdeo AG, Kale VM (2015). Comparative pharmacognostic and phytochemical investigation of two *Alpinia* species from Zingiberaceae Family. World J Pharm Res.

[12] Chudiwal AK, Jain DP, Somani RS (2010). *Alpinia galanga* Willd. An overview on phyto pharmacological properties. Indian J Nat Prod Res.

[13] Siringam K, Thongket T, Vajrodaya S, Mosaleeyanon K, Kirdmane C (2012). Optimization of air temperature and medium pH enhanced growths and 1′-Acetoxychavicol acetate (ACA) content of galangal (*Alpinia galanga*) plantlets *in vitro*. KMITL Sci Tech J.

[14] Tag H, Das AK, Loyi H (2007). Anti inflammatory plants used by the Khamti tribe of Lohit district in eastern Arunachal Pradesh, India. Nat Prod Rad.

[15] Madhuri S, Pandey G (2009). Some anticancer medicinal plants of foreign origin. Curr Sci.

[16] Girija TP, Rema SAB (2014). Comparative anatomical and histochemical characterization of the source plants of the Ayurvedic drug Rasna., inter. J. Herbal Med.

[17] Anonymous, Unani Pharmacopoeia of India. Government of India Ministry of Health and Family Welfare, Department of Ayurveda, Yoga & Naturopathy (AYUSH). New Delhi; 2007; 2(1): 67–68.

[18] International conference on harmonization (ICH) of technical requirements for the registration of Pharmaceutical for Human use. In: ICH harmonized tripartite guideline validation of analytical procedures: text and methodology Q2(R1); 2012.

[19] Anonymous. The United states pharmacopoeia, 31st Edition The national formulary, 26th edition, The United states pharmacopeia convention. Rockville; 2007.

[20] Anonymous, TLC Atlas of Chinese crude drugs in pharmacopoeia of the People’s Republic of China, Chinese Pharmacopoeia Commission People’s Medical Publishing House; 2009.

